# The evidence base for rotavirus vaccination in India: Current status, future needs

**DOI:** 10.1016/j.vaccine.2024.126551

**Published:** 2025-01-12

**Authors:** Niranjan Bhat, Elisabeth Vodicka, Allison Clifford, Kanduri Balaji Ananth, Ashish Bavdekar, Arup Deb Roy, Umesh Parashar, Jacqueline Tate, Pradeep Haldar, Gagandeep Kang

**Affiliations:** a Center for Vaccine Innovation and Access, PATH, 2201 Westlake Avenue, Suite 200, Seattle, WA 98121, USA; b Center for Vaccine Innovation and Access, PATH, 455 Massachusetts Ave NW, Washington, DC 20001, USA; c Center for Vaccine Innovation and Access, PATH, 15th Floor, Dr. Gopal Das Bhawan, 28 Barakhamba Road, New Delhi 110001, India; d KEM Hospital Research Centre, Vadu Rural Health Program, P.O. Vadu Budruk, Taluka Shirur, District Pune 412216, India; e JSI India, Plot No.5 & 6, LSC Shopping Complex, Nelson Mandela Marg, Vasant Kunj, New Delhi 110070, India; f Centers for Disease Control and Prevention, 1600 Clifton Rd. NE, Atlanta, GA 30333, USA; g Former Advisor (RCH), Ministry of Health and Family Welfare, Government of India, Delhi, India; h Division of Gastrointestinal Sciences, The Wellcome Trust Research Laboratory, Christian Medical College, Vellore, Tamil Nadu 632004, India

**Keywords:** Rotavirus, Diarrhea, Rotavirus vaccine, Vaccination, Vaccine impact, India

## Abstract

Rotavirus is a leading cause of severe diarrheal disease in infants and young children worldwide. Vaccination offers the best protection against this disease, and two rotavirus vaccines were developed in India and included in its routine immunization program. The Government of India's decision to adopt this intervention was supported by a solid base of evidence from clinical trials, as well as substantial research regarding rotavirus disease burden and the potential health and economic value of immunization. Following program implementation, multiple studies were initiated, including three evaluations of effectiveness and several investigations regarding intussusception. These additional data regarding vaccine impact, safety, and delivery from post-introduction evaluations in conditions of real-world use will further strengthen and sustain the immunization program. This manuscript evaluates the status of existing and forthcoming evidence regarding rotavirus vaccination in India through a literature review and consultation with relevant stakeholders. Studies evaluating vaccine impact, effectiveness, safety, health economics, and acceptability, as well as operational and programmatic research, were included in the review. Overall, we found that the evidence base did not contain any major gaps. Nevertheless, additional smaller-scale research studies would be valuable in providing a more complete picture of rotavirus vaccine performance and benefit. Documentation of India's experience with rotavirus vaccines may provide lessons learned for other countries in the Asia region, where rotavirus disease burden remains high, yet vaccine adoption has been slow, as well as for countries worldwide that may be considering implementation of the Indian-made rotavirus vaccines.

## Highlights

• Two rotavirus vaccines developed in India are included in its immunization program.
• We reviewed existing and forthcoming evidence on rotavirus vaccination in India.
• No major gaps found in the evidence base, supporting government's decision-making.
• Additional research on rotavirus vaccine performance and benefit may be valuable.

## 1 Introduction

Rotavirus is a leading cause of severe diarrheal disease in infants and young children worldwide. According to World Health Organization (WHO) estimates, 122,000 to 215,000 childhood diarrheal deaths due to rotavirus occurred annually worldwide between 2013 and 2017, with more than 90 % of all rotavirus-related deaths occurring in resource-poor countries of South Asia and sub-Saharan Africa. [1] India in particular has borne a substantial proportion of the world's rotavirus burden, as estimates from 2011 to 2013 indicated that approximately 872,000 hospitalizations and 78,000 deaths associated with rotavirus in children occurred in India each year during that period. A subsequent study using different methods estimated approximately 10,000 deaths in 2016, consistent with the overall global decline in pediatric rotavirus-associated mortality observed worldwide over time. [2,3]

Vaccination offers the best protection for infants against severe diarrhea and death from rotavirus infection, and several live, orally administered rotavirus vaccines are currently available for global use. [4] Of these vaccines, ROTAVAC® (Bharat Biotech) and ROTASIIL® (Serum Institute of India, Pvt. Ltd.) and their derivatives were developed in India using a 3-dose schedule and have been included in the country's Universal Immunization Programme (UIP). Both original vaccines achieved WHO prequalification in 2018 and are now being introduced in a number of countries worldwide. [5]

India's decision to introduce rotavirus vaccine was supported by an extensive base of evidence regarding rotavirus disease burden and vaccine performance. [6] However, with the introduction of any vaccine, additional data can help strengthen and sustain the immunization program by documenting impact, assuring safety, and improving delivery. This may even be more important for rotavirus vaccines given that the efficacy seen in the ideal conditions of a clinical trial could differ from the real-world performance in routine conditions and shifts in circulating strain distributions. Also, safety concerns related to intussusception (an adverse event associated with earlier rotavirus vaccines) could not really be examined in the pre-licensure trials in a meaningful way given the small sample sizes, so post-licensure monitoring is key. Assessing the cost, reach, health impact, and economic benefits of rotavirus vaccination in India should provide important evidence to maintain strong long-term support, optimize its value, and share learnings with the global community. Documenting India's experience may be particularly helpful for other countries in Asia and the Pacific region, where rotavirus vaccination implementation has been slow and disease burden remains high, [7] as well as for countries worldwide, including more than ten in Africa, that are expecting to implement these Indian-made rotavirus vaccines.

Towards this end, we evaluated the status of existing and forthcoming evidence regarding the health impact of rotavirus vaccines in India, including conducting a literature review and consulting with relevant stakeholders. These stakeholders included representatives from the Government of India, research institutions, non-governmental organizations, and rotavirus vaccine manufacturers. In this review, we summarize our findings, with the goal of identifying critical gaps in the evidence base, proposing potential approaches to address them, and considering ways in which those efforts could be implemented.

## 2 Introduction of rotavirus vaccine in India

**Fig. 1:**
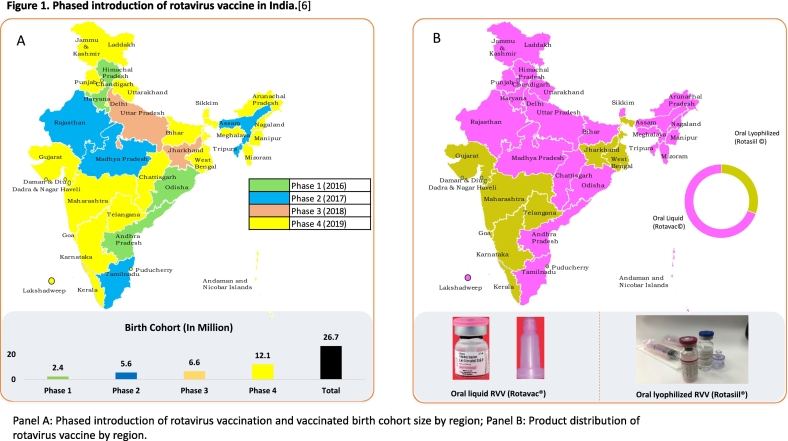
Phased introduction of rotavirus vaccine in India. [6]. Panel A: Phased introduction of rotavirus vaccination and vaccinated birth cohort size by region; Panel B: Product distribution of rotavirus vaccine by region.

With a birth cohort of 26.7 million, rotavirus vaccine roll-out in India was divided into several phases (Fig. 1). The first phase introduced rotavirus vaccine in March 2016 in four states: Orissa, Andhra Pradesh, Haryana, and Himachal Pradesh. The second phase began in February 2017 and added five states: Assam, Tripura, Rajasthan, Madhya Pradesh, and Tamil Nadu. The third phase added two more states, Jharkhand and Uttar Pradesh in 2018. [6] In the accelerated fourth phase in 2019, the government decided to complete the pan-India rollout as part of their “100 days” agenda, with rotavirus vaccine rolled out in the remaining 25 states and union territories. The nationwide rollout was completed in April 2020. [8] After being fully integrated into the national immunization program, more than 60.5 million doses were delivered across India between November 2019 and October 2020. [9]

The extensive pre-introduction activities conducted by India's Ministry of Health and Family Welfare (MoHFW) in each state included training material and communication plan development, cold chain space assessment, and training of healthcare workers. Immunization worker training packages were tailored to the presentation of each of the two Indian vaccines in a cascade format, initially targeting national and state officials who would then train healthcare workers at the district and block levels. More than two million healthcare workers were trained for the vaccine rollout through a cascaded training model supported by all immunization partners. [8]

Having ultimately achieved more than 90 % coverage, the rollout was a success story overall, though some challenges were encountered during the process. For instance, the overlapping introduction of two very different rotavirus vaccine products in different geographies led to practical problems in ensuring homologous follow-on doses in the absence of vaccine interchangeability data/guidelines, particularly in areas of high migration during the initial stages of introduction. Data from the ongoing interchangeability studies were not yet available at that time. In terms of product-specific challenges, healthcare workers expressed concerns regarding the potential effect of multiple freeze-thaw cycles on the stability of ROTAVAC (a frozen liquid formulation) and ROTASIIL (a multi-component lyophilized formulation), which required additional time to adjust to the relatively more complex storage and labor demands. This was, however, overcome rather quickly with additional trainings and interchangeability guidelines issued by the MoHFW after the National Technical Advisory Group on Immunization (NTAGI) approved the data from interchangeability studies. These guidelines were issued in a graded manner, first allowing vaccine interchangeability for migration between different states, and later within the states when the liquid formulation of ROTASIIL replaced the lyophilized version. Like the rest of the world, India experienced the challenges posed by the COVID-19 pandemic on routine immunization, which significantly affected coverage especially during the early lockdown phases. Over the subsequent months, as the lockdown restrictions were gradually relaxed and immunization services resumed, overall immunization coverage returned almost to baseline. Rotavirus vaccine coverage in particular has experienced a steady rise over time despite the pandemic, reaching an estimated coverage of 82 % in 2020 and 92 % in 2022. [10]

## 3 Vaccine impact and effectiveness

Vaccine impact is a measure that can provide an aggregate assessment of vaccine effectiveness that accounts for multiple contextual factors, including underlying disease prevalence, vaccine performance, immunization coverage, and implementation efficiency. Analyses of impact are typically conducted by examining trends in disease surveillance before and after vaccine implementation. Therefore, the availability of data prior to introduction is critical to making a valid assessment of a vaccination program's success.

In the case of India, the decision to launch a national rotavirus vaccination program was based on a solid foundation of surveillance data regarding rotavirus hospitalizations that was geographically distributed and spanned several years. For instance, the National Rotavirus Surveillance Network, involving 28 hospital sites and 11 laboratories across India, documented an overall rotavirus prevalence of 36.3 % among children younger than five years of age hospitalized for diarrhea. [11] Another network comprising seven cities in southern and northern India observed a rate of 35.5 % in this same age group. [12] Initial trends from continued surveillance following vaccine introduction have indicated a reduction in rotavirus hospitalization rates in sites where ROTAVAC had been launched [[13], [14], [15], [16], [17], [18], [19], [20], [21], [22], [23], [24], [25], [26], [27], [28]] and are corroborated by indirect analyses of impact using household survey data. [29] These studies, therefore, have served to verify the benefits and track program performance over time, data that other countries may find critical in making their own decisions about rotavirus vaccine product options.

Nevertheless, measuring impact can often be challenging due to various factors, such as secular changes in disease incidence that may complicate interpretation and assessment of immunization impacts on mortality. This can become increasingly difficult to measure as health conditions improve more generally. Moreover, rotavirus surveillance data from 2020 onward may have reflected impacts of the COVID-19 pandemic. As noted above, rotavirus vaccination coverage increased steadily despite multiple waves of COVID-related illness in the country. However, the overall effects of the pandemic on rotavirus disease may have been complex, as COVID-related burdens on the healthcare system may have displaced rotavirus-related hospitalizations, while social distancing may have altered healthcare-seeking behavior or decreased transmission of diarrheal diseases at the time. [30]

Thus, in addition to evaluations of vaccine impact, existing and forthcoming evidence regarding rotavirus vaccine effectiveness can provide increased understanding of the performance of this intervention in real-world conditions. When considering these data in the Indian context, it is important to consider estimates of rotavirus vaccine efficacy and effectiveness from other parts of the world, and note the significantly reduced performance of these vaccines in lower-resource settings compared to higher-income settings. [[31], [32], [33]] This differential in outcomes highlights the importance for countries to generate local, real-world data, if feasible, to confirm the benefit of rotavirus vaccine for their own populations. To that end, three studies of rotavirus vaccine effectiveness were initiated in India between 2016 and 2020.

**Fig. 2:**
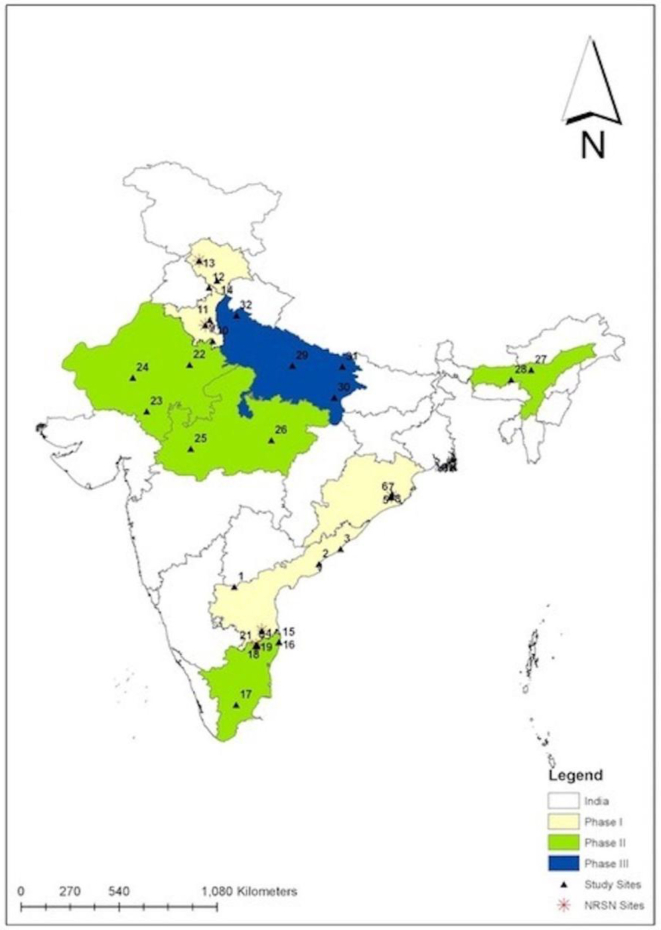
Location of surveillance sites in states introducing ROTAVAC. [33].

Source: [33]

The first study involved the establishment of active surveillance for rotavirus-associated acute gastroenteritis hospitalizations among children younger than five years of age across more than 30 clinical sites located in Indian states selected for initial introduction with ROTAVAC, [34] many of which were already participating in the surveillance networks mentioned above (Fig. 2). Embedded within this activity was a vaccine effectiveness evaluation using a test-negative, case-control study design. Enrollment in this study began in 2016 and was carried out in nine states in a staged fashion that tracked with the rollout of ROTAVAC. The active phase of the study was completed in 2020, with approximately 24,000 children enrolled in the surveillance component and approximately 2000 cases and 6000 controls in the effectiveness evaluation; results are forthcoming.

Two additional studies, following a similar design but assessing the effectiveness of ROTASIIL, have been initiated. As with the first study, these smaller investigations embedded a test-negative, case-control evaluation within a larger surveillance study, each targeting a sample size of approximately 200 to 450 cases. One study, conducted in Jharkhand, the initial state for the introduction of ROTASIIL, was launched in July 2019, and after experiencing some delays due to the COVID-19 pandemic, has completed enrollment. [35] The second study employs the same design and target sample size across four additional states using ROTASIIL: Kerala, Karnataka, Maharashtra, and Gujarat. Due to COVID-related delays, this study began in March 2020 and has also completed enrollment. Results from both studies are forthcoming.

These three studies will provide core data to create a basic picture of the clinical performance of rotavirus vaccines in India. Beyond these results, future research may be considered to address remaining gaps in the evidence regarding the current vaccine regimens, including assessment of vaccine effectiveness against additional outcomes, such as severe disease or all-cause diarrhea; genotype-specific effectiveness and strain evolution, with already some indication of a shift in genotype predominance from G1P [8] to G3P [8] following vaccine introduction [[36], [37], [38]]; effectiveness in special populations, such as HIV-exposed (if feasible) and malnourished children; and evaluation of herd protection. Studies going forward could also seek to improve upon the existing approaches to improve efficacy, for instance through evaluation of alternative schedules or research into potential effect modifiers of vaccine-induced protection, such as the gut microbiome, human milk oligosaccharides, and histo-blood group antigens. In addition, some evidence suggests that co-infection of rotavirus with other enteric pathogens may modify vaccine effectiveness, [39] although this has not been seen consistently [40] and will require further study.

## 4 Vaccine safety

Safety has been a critical issue in the adoption of rotavirus vaccines worldwide, focused primarily on the potential for an increased risk of intussusception (IS) following vaccination. Large studies in several high- and middle-income countries have detected a small increased risk of IS associated with RotaTeq® (Merck & Co.) and ROTARIX® (GlaxoSmithKline [GSK]), with an estimated 1 to 6 excess cases per 100,000 vaccinated infants within the 7 to 21 days following any vaccine dose, and the greatest risk in 1 to 7 days after the first dose. [1] However, no such additional risk was observed in a study conducted across several African nations, [41] indicating that this concern may not be uniform across different geographies or child mortality strata. Accordingly, investigating the risk of IS has been a priority in the development and implementation of ROTAVAC and ROTASIIL.

Given that less than 10,000 infants were included across each of the two Phase 3 clinical trials of the Indian vaccines, the pre-licensure data that established their efficacy was insufficient to assess for a risk of IS of the low magnitude seen with the other rotavirus vaccines as described above. [42,43] In advance of the rollout of ROTAVAC in India's UIP, policy-making bodies, including the NTAGI and the WHO, called for further examination of this issue during vaccine implementation. While data regarding background rates of IS in India were available, [44] analytic studies that could examine the specific risk of vaccination were needed. In response, three large, geographically distributed studies were conducted in the areas of vaccine introduction. Implemented through collaborations among academic institutions, non-governmental organizations, and governmental bodies, these studies all employed the established self-controlled case series (SCCS) method to detect any potential increase in IS risk following rotavirus vaccination.

The first study, led by the Centre for Health Research and Development, Society for Applied Studies (CHRD SAS), was conducted in parallel with a pilot introduction of ROTAVAC in Maharashtra, Himachal Pradesh, and Tamil Nadu. Beginning in late 2015, this study enlisted a network of 35 sentinel hospitals to conduct enhanced passive surveillance with central level review. By study completion at the end of 2019, 151 IS cases were identified, and SCCS analyses showed no increased risk of IS following immunization. [45]

A second study, led by the Translational Health Science and Technology Institute (THSTI), conducted active IS surveillance in 27 sentinel hospitals located in ten states. Beginning in four states in April 2016, the network expanded in a phased manner in parallel with ROTAVAC introduction, and the study was completed in mid-2018, ultimately analyzing a total of 589 children with IS. As with the first study, this investigation found no evidence of an association between rotavirus vaccination and IS by SCCS analysis, a finding corroborated by a concurrent case-control sub-analysis. [46]

The third study, led by the INCLEN Trust International, enlisted 23 tertiary care hospitals across all regions of India to conduct retrospective and prospective surveillance for IS from April 2016 to September 2017. [[47], [48], [49]] Among these sites where ROTAVAC was introduced, the study team identified 670 children with IS within the target age range, among whom 311 had vaccination information and required surgical or radiological intervention. Of this smaller group, 52 had confirmed receipt of rotavirus vaccine and were included in the analysis, which demonstrated no increased risk of IS due to vaccination. Subsequent descriptive case series in the post-introduction period have provided additional reassurance that IS rates have not increased following widespread rotavirus vaccine uptake. [[50], [51], [52], [53]]

Overall, these initiatives established a strong base of evidence indicating no increased risk of IS following rotavirus vaccination occurred in India, suggesting that additional large-scale studies are not needed. However, as all of the investigations took place in settings that use ROTAVAC, and the risk profile could differ for each vaccine, field data specifically evaluating ROTASIIL is important to have as well. Fortunately, the two surveillance and effectiveness studies of ROTASIIL in the five states described above are both associated with IS surveillance activities, with the latter study having identified well over its target of 600 cases by study closure.

## 5 Health economics

In addition to considerations regarding vaccine effectiveness, safety, and operational feasibility, economic evidence can support value-based vaccine program investments, inform product choice decision-making, and help countries plan for program sustainability over time. Such data include modeled or empirical evidence on the health impact and cost-effectiveness of vaccination, in addition to relevant data on financial and economic costs of vaccine introduction, recurrent costs of routine immunization, and costs of treatment for rotavirus-related diseases averted by vaccination.

As shown in Table 1, Table 2, Table 3, published data already exist on costs and health economic impact of rotavirus vaccination in India, although most studies were specific to ROTAVAC (*N* = 5) [2,[54], [55], [56], [57]] and relatively few assessed ROTASIIL (*N* = 2) [54,58]. Cost-effectiveness studies consistently suggest that universal vaccination against rotavirus among infants ranges from cost-saving to highly cost-effective health investment for the Indian government. [[54], [55], [56], [57], [58], [59], [60]] Several national and sub-national studies have also been conducted evaluating the costs of treatment for rotavirus-related diseases, such as diarrhea. These data indicate a broad and supportive economic evidence base for rotavirus vaccination in India.

**Table 1:** Published studies on the health and economic impact of rotavirus vaccine delivery in India.

**Author, year**	**Model approach**	**Perspective(s)**	**Vaccine(s) evaluated**	**Comparator**	**Key findings**⁎
Constenla, 2019 [56]	Economic impact model	Societal	Generic rotavirus vaccine	No vaccination	Public Sector Results Under-5 annual deaths averted nationally: 21,236-26,545 Total cases averted nationally: 713,226-891,532 Total costs averted nationally: $232,341,801–$290,427,263 Private Sector Results Under-5 annual deaths averted nationally: 21,904-27,380 Total cases averted nationally: 735,672-919,589 Total costs averted nationally: $1,175,740,370-1,469,675,463
Debellut, 2019 [51]	Proportional outcomes model *(global estimates with India-specific estimates also provided)*	Government and societal	50 % ROTAVAC; 50 % ROTASIIL	No vaccination	Total cases averted over 10 birth cohorts: 46,083,379 Total deaths averted over 10 birth cohorts: 106,007 Cost per DALY averted from government perspective: $665 (95 % credible range: $484–$845)
Lee, 2017 [55]	Economic impact model	Government and program	Thermostable vaccine (assumed ROTASIIL)	Non-thermostable vaccination	Range from cost-saving to $1221/DALY per 100 people Cost per dose: $11.60
Rose, 2017 [52]	Dynamic simulation model	Government; family; societal	ROTAVAC	No vaccination	$63/DALY Averted Cost per dose: $1.12 (0.56, 2.23)
John, 2014 [2]	Economic impact model	Societal	ROTAVAC	No vaccination	Total cost of rotavirus immunization program in 2011 (birth cohort of 27,098,000): $86,668,129 compared to estimated rotavirus-related hospitalization costs of $92 M ($62-$130 M) and outpatient visit costs of $101 M ($68-$143 M). Cost per dose: *$0.94*
Megiddo, 2014 [53]	Cost-utility analysis	Program	Vaccine 116E (assumed ROTAVAC)	Modeled addition of rotavirus vaccine to existing EPI portfolio at varying coverage	All interventions modeled were cost saving Cost per dose: NR
Esposito, 2011 [57]	Cost-utility analysis	Health system	Vaccine performance data based on RotaTeq trials in Bangladesh and Vietnam	No vaccination	$27/DALY Averted; $831/life saved; Cost per dose: $1.25 ($0.18–$8.78)
Rose, 2009 [54]	Cost-utility analysis	Government; household; societal	ROTAVAC	No vaccination	$63/DALY Averted Cost per dose: $1.12 ($0.56–$2.23)

⁎ Reported costs have been converted to 2021 U.S. Dollars (USD)s using World Bank Gross Domestic Product deflators and World Bank exchange rate (local currency unit to USD) to allow for comparison across studies. [58,59].

**Table 2:** Published studies on cost of delivery estimates for rotavirus vaccination in India.

**Author,** **year**	**Vaccine evaluated**	**Region**	**Reported costs of administration**⁎	**Cost components**
Rose, 2017 [52]	ROTAVAC	National	$0.62 ($0.42–$0.82) per dose	Administration, transportation, storage *(Note: Costs calculated based on non-ROTAVAC-specific data*⁎⁎*)*
John, 2014 [2]	ROTAVAC	National estimates based on data from Vellore and Tamil Nadu	≤ $0.09 per dose	Incremental delivery costs of adding ROTAVAC to EPI; cost categories not specified
Megiddo, 2014 [53]	116E (assumed ROTAVAC)	National estimates stratified by rural/urban	$26.17 per child (baseline DPT3, no rotavirus); $27.34 ($19.14–$35.54) for baseline DPT3 plus rotavirus immunization at DPT3 coverage level; $31.41 ($21.99–$40.83) for baseline DPT3 plus rotavirus immunization under assumption of increased coverage rates.	Total routine immunization costs: costs for vaccines, personnel, vehicles and transportation, cold chain equipment and maintenance, and program and other recurrent costs *(Note: Did not distinguish ROTAVAC-specific delivery costs from total immunization costs but presented total costs with addition of ROTAVAC under varying coverage scenarios).* Calculated incremental costs for addition of rotavirus immunization range from $1.16 to $5.23 under the two scenarios with addition of rotavirus to DPT3 regimen.

⁎ Reported costs have been converted to 2021 U.S. Dollars (USD)s using World Bank Gross Domestic Product deflators and World Bank exchange rate (local currency unit to USD) to allow for comparison across studies. [58,59].

⁎⁎ Atherly et al. [70]; Esposito et al. [57].

**Table 3:** Published studies on cost of treatment or cost of illness estimates related to rotavirus disease in India.

**Author,** **year**	**Region**	**Perspective**	**Types of costs**	**Location of services** **costed**	Results*	**Variation assessed**
Constenla, 2019 [56]	National, plus state-level estimates for Bihar, Delhi, Maharashtra, Tamil Nadu	Societal	Direct medical; non-medical; indirect *Currency: 2018 USD*	Inpatient; outpatient	Inpatient cost per bed day – public sector: $61 ($48–$73); Inpatient cost per bed day – private sector: $264 ($211–$318) Outpatient visit cost – public sector: $2 (rural); $4 (urban, range: $3–$9) Outpatient visit cost – private sector: $9 (rural, $6–$10); $10(urban, range: $6–$43)	State-level variation; private vs. public facilities
Zimmermann, 2019 [60]	National	Household	Out-of-pocket direct medical costs, non-medical, indirect	Inpatient; outpatient	Unadjusted total out-of-pocket costs (US$ 2012): Median: $8.10; Mean: $13.34	Age-strata; disease severity
Jacob, 2016 [61]	Vellore	Payer; societal	Direct medical; indirect	Inpatient; outpatient	Median outpatient costs: Rs 590 (direct) and Rs 190 (indirect); Median inpatient costs: Rs 7258 (direct) and Rs 610 (indirect) *Currency year not specified; therefore, these costs have not been adjusted to 2021 USD.*	Under 5
Mathew, 2016 [62]	Delhi	Payer	Direct medical	Inpatient	Median rotavirus costs: Rs 17,941–50,663 (Pediatric Intensive Care Unit); Rs 5787–10,088 (High Dependency Unit) *Currency year not specified; therefore, these costs have not been adjusted to 2021 USD.*	Under 5; rotavirus v. no rotavirus; care setting; periods of episodes (2005–2008 and 2012–2014)
Kumar, 2014 [63]	Haryana	Patient (out-of-pocket)	Direct medical	Inpatient; outpatient	Average out-of-pocket cost per episode: Rs 444 (95 % CI: 299–589); Outpatient setting: Rs 203 (95 % CI: 188–232); Inpatient setting: 5734 (95 % CI 3336-8131) *Currency year not specified; therefore, these costs have not been adjusted to 2021 USD.*	Under 2
John, 2014 [2]	National	Payer	Direct medical	Inpatient; outpatient	Median direct costs: $159 (tertiary care); $132 (secondary); $36 (primary care); $4 (outpatient)	Under 5; facility type; region
Namjoshi, 2014 [64]	National	Payer	Direct medical	Inpatient; outpatient	Mean direct costs: 3177, SD: 4789 (RV-positive); 1787, SD: 4262 (RV-negative) *Currency year not specified; therefore, these costs have not been adjusted to 2021 USD.*	Under 5; rotavirus v. no rotavirus; private facilities only
Patel, 2013 [65]	Kuala Bandar in Mumbai	Household;	Direct medical; indirect	Not specified	Mean household cost per diarrheal episode: Rs 409 *Currency year not specified; therefore, these costs have not been adjusted to 2021 USD.*	Under 5; urban slum only
Sowmyanrayan, 2012 [66]	Vellore, Pune, Kolkata, New Delhi	Payer; Household	Direct medical; non-medical; indirect (Note: Utilization patterns were inferred)	Inpatient; outpatient	Mean cost per hospitalized episode: $53.75 (rotavirus-positive); $66.05 (rotavirus-negative) *Currency year not specified; therefore, these costs have not been adjusted to 2021 USD.*	Under 5; rotavirus v. no rotavirus; Facility Type
Rheingans, 2012 [67]	National level costs of treatment for diarrheal illness	Household	Direct medical; non-medical; indirect	Inpatient; outpatient	All care-seeking: $4.02; Incurring treatment costs: $6.40	Under 5; household income levels; formal vs. informal costs; all care-seeking vs. incurring any treatment costs
Tate, 2009 [68]	National estimates extrapolated from regional data (Vellore and Pune)	Societal	Direct medical; non-medical; indirect	Inpatient; outpatient	Weighted median cost per hospitalization: $90.10; Weighted median cost per outpatient visit: $4.83	Under 5; facility type
Mendelsohn, 2008 [69]	Vellore	Societal; household	Direct medical; non-medical; indirect	Inpatient; outpatient; emergency room costs	Societal costs per episode: $80.80 (referral hospital); $40.60 (community hospital) *Currency year not specified; therefore, these costs have not been adjusted to 2021 USD.*	Under 5; rotavirus v. no rotavirus; facility type

***Where possible, reported costs have been converted to 2021 U.S. Dollars (USD)s using World Bank Gross Domestic Product deflators and World Bank exchange rate (local currency unit to USD) to allow for comparison across studies. [58,59] For studies that did not report a clear currency year, we have chosen to report the originally reported costs from those studies without conversion to avoid subjective interpretation of the reported results.

Although rotavirus vaccine has already been introduced in India and the economic evidence is compelling, additional financial and economic data may be valuable for future decision-making, particularly related to the potential impact of product switching as new products come under consideration, program delivery, and vaccine financing over time. Additionally, subnational cost data remains an economic evidence gap that could inform local decision-making. Many studies extrapolated regional-level data to the national level or estimated costs based on data from a small number of regions. However, as India is a large, populous, and diverse nation, data from one region might not be representative for other regions and additional sub-national analyses may be beneficial, particularly with regards to the cost of vaccine delivery, as programmatic issues around presentation and wastage may differ across geographies. Further, new cost of delivery analyses specific to ROTASIIL would be useful given the substantially different presentation and logistical requirements of this product compared to ROTAVAC. In addition, operational challenges, as discussed below, often have downstream cost and resource allocation implications that may not be consistently quantified in the existing economic literature. Assessment of the economic effects of indirect (herd) protection for unvaccinated age groups due to decreased rotavirus transmission could provide a more complete picture of the benefits of vaccination.

A more recent area of research focuses on the question of whether, in overcrowded resource-limited settings, a vaccine-related reduction in diarrheal hospitalizations may open up beds for patients who were otherwise getting turned away. [61] Future research on operational capacity impacts of averting hospitalizations associated with rotavirus and other vaccine-preventable diseases (and potential impacts of the COVID-19 pandemic) may provide broader understanding of the indirect benefits to the health system yielded from vaccine investments. Finally, examination of immunization delivery costs in the Indian private sector (due to the potential for differential uptake among vaccine products) and comparative cost of delivery analyses with alternative interventions, such as in sanitation and hygiene, may be of interest in the future.

## 6 Vaccine acceptability

Data on attitudes and the acceptability of vaccines, including studies among policy- and decision-makers involved in the national immunization program, physicians and other healthcare workers, and parents of infants and young children can help support the sustainability of any specific immunization program. In India, vaccines have generally enjoyed strong support across all of these stakeholder groups, and parents in particular tend to feel that childhood vaccines are important, effective, and a good way to protect against disease. [62] Nonetheless, recent evidence suggests that parental hesitancy about childhood vaccines is emerging as a reason for non-vaccination in India. [63]

While some national and global data specifically on rotavirus vaccine acceptability do exist, little primary research on this topic has been conducted in India. A 2018 systematic review offers lessons learned from other countries on the acceptability of rotavirus vaccine and potential implications for the Indian context. [64] In addition, earlier studies on parental and private-sector attitudes towards rotavirus and other childhood vaccines, such as those against pneumococcus, *Haemophilus influenzae* type b, and influenza exist as well. [[65], [66], [67], [68]] The relevance of research conducted in other countries, or even globally, on attitudes regarding vaccines more generally may ultimately be applicable to the Indian rotavirus vaccine context.

Our consultations with stakeholders identified new areas where additional evidence could be valuable including studies among the various stakeholder groups, as described above, with a focus on concerns and barriers unique to rotavirus vaccines, such as their oral administration and the Indian context. In the Indian public sector, the main difficulties around rotavirus vaccine acceptability and narrative control may be with the healthcare workers rather than parents (e.g., the risk of intussusception associated with rotavirus vaccines has not been a significant concern for the general public). Other potential gaps in acceptability research include the assessment of attitudes towards rotavirus vaccine among the private sector, and among pediatricians, who are classified as specialists in India. Studies evaluating the stratification of vaccine hesitancy according to socio-economic levels or other social determinants of health may also be of interest, although not necessarily specific to rotavirus vaccines.

## 7 Operational and programmatic research

While the Government of India would play the primary role in strengthening rotavirus immunization delivery through program improvement efforts, researchers can support this work through activities that emphasize hypothesis-driven analytical evaluation. For instance, multiple sources of data regarding immunization systems in India, including administrative data, coverage surveys, monitoring reports, targeted assessments, and surveillance activities, exist that could provide opportunities for operational research. State-level rotavirus vaccine coverage estimates indicate that a majority of states where the vaccine had been introduced remain in the 60 to 80 % range, although rates were higher in a limited number of areas. [9] Much of the gap in coverage can be attributed to initial operational challenges (e.g., tight timelines for training materials, concerns about interchangeability of products, and product-specific challenges), as these rates ultimately improved as the rollout progressed, and comparisons with global rotavirus vaccination rates over time have suggested that achieving a moderate coverage level at first, followed by a slow increase, is not uncommon.

Themes in vaccine implementation-focused research often center on examining ways to improve coverage data, ranging from data abstraction from established automated electronic systems to more resource-intensive special studies. Sources of coverage variability can be assessed across multiple operational indicators, including vaccination coverage, wastage rates, and adverse event reporting, which can lead to the identification of drivers of differential coverage rates by district level or by specific vaccine. For instance, in the early months of the rollout, rotavirus vaccine coverage rates were typically lower than coverage rates for the injectable pentavalent vaccine (DTP-Hib-HepB). This prompted questions of whether the difference was specific to rotavirus vaccine, associated with other characteristics more generally, or simply due to introduction-related issues that would be resolved over time, such as unfinished training, more intensive supervision needs, and stockouts due to initial supply-demand mismatches. Additional research might examine the interplay among the number of doses per vial, immunization session size, cold-chain storage capacity, and wastage rates. [69]

The strategy of providing different vaccine products by state combined with the inevitable occurrence of interstate migration points to the need to examine the safety and effectiveness of mixed-dose regimens, which would support the interchangeability of the vaccines. Guidance based on limited data has been issued by the WHO in support of the interchangeability of the Merck and GSK rotavirus vaccines, [70] however this advice does not currently include the Indian-manufactured vaccines. To address this, an open-label, randomized controlled trial involving almost 2000 participants was conducted in Maharashtra and West Bengal during 2019 to 2020. [71,72] The evaluation compared seroresponse rates and safety among different combinations of ROTAVAC and ROTASIIL, ultimately showing that the vaccines can indeed be used interchangeably.

Data from existing national and sub-national electronic systems, such as the Health Management Information System (HMIS) [73], the Mother Child Tracking System (MCTS) [74], and the electronic Vaccine Intelligence Network (eVIN) [75], could also be accessed to conduct larger-scale analyses of clinical and logistical issues without requiring substantial additional resources. Ultimately, while conducting research around rotavirus vaccine implementation may be valuable for the ongoing success of the program, most questions are not specific to rotavirus vaccines, but applied across all vaccines in the routine schedule, and the primary driver for work in this area will likely be the government, under the auspices of monitoring and evaluation activities.

## 8 Conclusions

Initial support for rotavirus immunization and the decision to introduce this intervention in India were built on a solid foundation of research that documented the public health burden of rotavirus disease, estimated the potential value of immunization, and facilitated the development of two licensed vaccines within the country itself. The introduction of rotavirus vaccine in India, involving one of the largest immunization cohorts in the world, was an undertaking of tremendous scale and significant complexity. The Government of India has now expanded the program nationwide, with coverage of more than 80 % in a majority of states. [10]

**Fig. 3:**
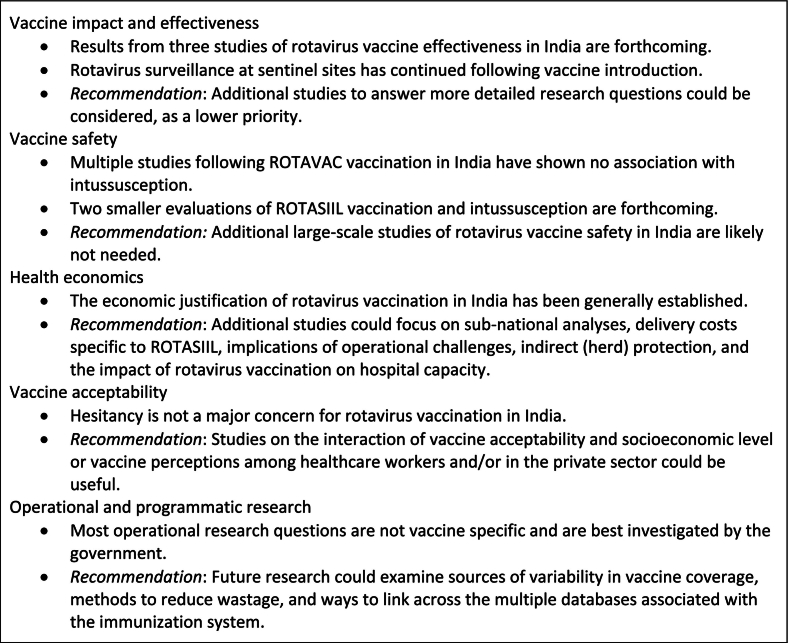
Key concepts and recommendations.

A commitment to policy-making that is grounded in evidence led to several major ongoing or completed studies occurring through the collaboration of governmental bodies, academic institutions, and non-governmental organizations. These evaluations promise to provide useful and credible information regarding the safety, effectiveness, and public health impact of the two Indian rotavirus vaccines, but gaps in the evidence base nevertheless remain (Fig. 3).

While much of the work assessing the risk of intussusception has been completed and results have been disseminated, additional time is needed for effectiveness evaluations to be completed. In addition, impact analyses, while promising to date, will certainly be strengthened from additional years of surveillance data to confirm that current trends are sustained. Nevertheless, the anticipated evidence base in support of rotavirus vaccination in India will not contain critical gaps, nor are there major concerns or barriers regarding rotavirus vaccine implementation that require intensive resources. As a result, new research does not need to be large-scale, but will still be valuable in providing a more complete picture of rotavirus vaccine performance and benefit. This is particularly true for ROTASIIL, which was introduced after ROTAVAC and in fewer states. Research on operational, safety, and acceptability issues may be considered, albeit are lower in priority.

Additional research would be important to sustain, improve, and document the benefit of rotavirus vaccines. Furthermore, the availability of the Indian vaccines to other countries at a comparatively lower cost adds increased choice and supply to the global market. Policy-makers worldwide would therefore benefit from these data from India to support their decisions to introduce or switch to these vaccines.

## Funding statement

This work was supported by the 10.13039/100000865Bill & Melinda Gates Foundation (Grant number OPP1016899). Under the grant conditions of the Foundation, a Creative Commons Attribution 4.0 Generic License has already been assigned to the Author Accepted Manuscript version that might arise from this submission.

## Disclaimer

The findings and conclusions in this report are those of the authors and do not necessarily represent the official position of the Centers for Disease Control and Prevention (CDC).

## CRediT authorship contribution statement

**Niranjan Bhat:** Writing – review & editing, Writing – original draft, Methodology, Funding acquisition, Conceptualization. **Elisabeth Vodicka:** Writing – review & editing, Writing – original draft. **Allison Clifford:** Writing – review & editing, Writing – original draft, Conceptualization. **Kanduri Balaji Ananth:** Writing – review & editing, Project administration, Conceptualization. **Ashish Bavdekar:** Writing – review & editing, Writing – original draft. **Arup Deb Roy:** Writing – review & editing, Writing – original draft. **Umesh Parashar:** Writing – review & editing, Conceptualization. **Jacqueline Tate:** Writing – review & editing, Conceptualization. **Pradeep Haldar:** Writing – review & editing, Conceptualization. **Gagandeep Kang:** Writing – review & editing, Conceptualization.

## Declaration of competing interest

The authors declare the following financial interests/personal relationships which may be considered as potential competing interests: Niranjan Bhat reports financial support was provided by Bill & Melinda Gates Foundation. Elisabeth Vodicka reports financial support was provided by Bill & Melinda Gates Foundation. Allison Clifford reports financial support was provided by Bill & Melinda Gates Foundation. Kanduri Balaji Ananth reports financial support was provided by Bill & Melinda Gates Foundation. If there are other authors, they declare that they have no known competing financial interests or personal relationships that could have appeared to influence the work reported in this paper.

## Data Availability

No primary data were used for the research described in the article.
